# Tennis Courts in the Human Body: A Review of the Misleading Metaphor in Medical Literature

**DOI:** 10.7759/cureus.21474

**Published:** 2022-01-21

**Authors:** Amogh Ananda Rao, Smilee Johncy

**Affiliations:** 1 Internal Medicine, Jagadguru Jayadeva Murugarajendra (JJM) Medical College, Davangere, IND; 2 Physiology, Jagadguru Jayadeva Murugarajendra (JJM) Medical College, Davangere, IND

**Keywords:** chaos theory, fractals, endothelium, villi, alveoli, tennis courts

## Abstract

Medical literature is home to fancy descriptions, poetic metaphors, and ingenious comparisons. However, some comparisons can disguise the knowledge gap. Large surfaces in the human body, like the alveolar surface for gas exchange, villi for food absorption, and the endothelial lining of blood vessels, are frequently compared to a “tennis court.”

This narrative review explores this metaphor in detail, the discrepancies and factual inaccuracies across medical literature. It highlights the inappropriate use of Euclidean geometry and introduces fractal geometry, a language to define roughness.

## Introduction and background

Medical literature is home to fancy descriptions, poetic metaphors, and ingenious comparisons. No physician can relish anchovy sauce without being reminded of the pus in amebic liver abscess. The dark sky with bright twinkling stars reminds a radiologist of hepatic parenchyma and a pathologist of Burkitt lymphoma [1]. Poetic comparison evokes the reader’s imagination and ingrains itself in the long-term memory. Nevertheless, such comparisons can sometimes mask the gap in knowledge or understanding. One such intriguing instance encountered repetitively in literature is likening large surface areas to tennis courts. The surface area of the lungs available for gas exchange, the intestinal villi for the absorption of food particles, and the endothelial lining of blood vessels are all compared to the size of tennis courts.

**Figure 1:**
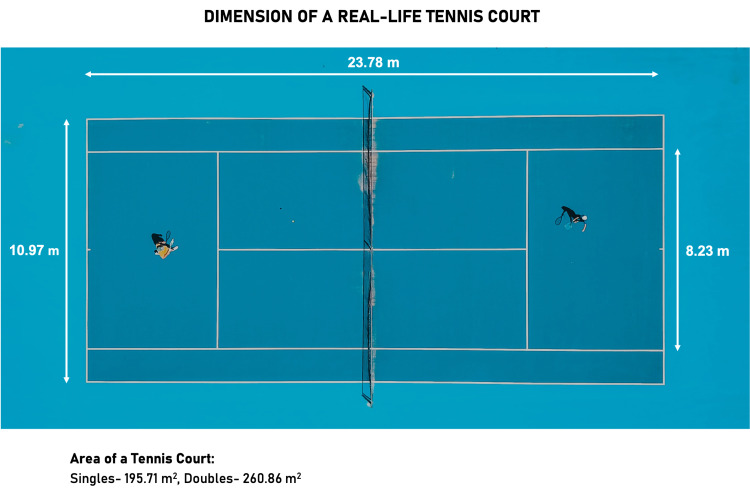
The dimensions of a real-life tennis court. The actual dimensions of a tennis court are 195.71 m^2^ for a match of singles and 260.86 m^2^ for doubles games [3].

The disparity in the estimated values of physiological surface areas and the discordance with the real dimensions of a tennis court is particularly interesting and worth investigating. The inconsistency of estimates is so stark that a popular textbook, “Berne & Levy Physiology,” provides two different estimates for the surface area available for gas exchange in two different chapters. It is approximated to be 85 m^2^ in Chapter 20 and 70 m^2 ^in Chapter 22; a comparison to tennis courts is drawn both times [2]. However, the actual dimensions of a tennis court are 195.71 m^2^ for a match of singles and 260.86 m^2^ for doubles games (Figure 1) [3]. Neither of the comparisons, even approximately, equates to the area of a real tennis court.

**Figure 2:**
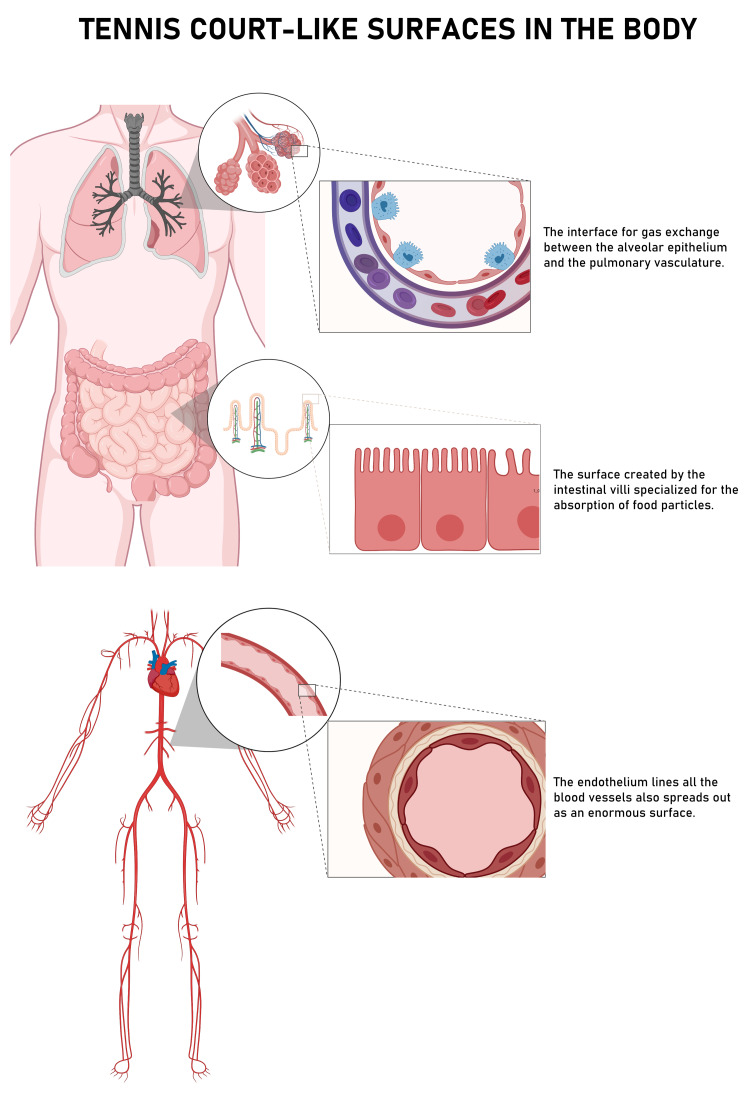
Surfaces in the human body commonly likened to tennis courts.

Figure 2 illustrates the three widely used instances of this metaphor in the medical literature. This narrative review explores this phenomenon and deconstructs the discrepancies in these physiological mismeasurements. It also addresses the inappropriate use of Euclidean geometry in describing shapes that are irregular or fractal. In the latter half, the article provides an intuitive introduction to fractal geometry as a language to describe roughness and irregularity.

## Review

We performed a cursory search on PubMed and Google Scholar for the term “tennis courts,” and selected relevant articles. Besides, we also conducted an in-text search for “tennis courts” within popular textbooks. The sentences comparing body surfaces to tennis courts were enlisted verbatim and referenced.

The alveoli

Historically, numerous experiments have tried to square in on the exact surface area available for gas exchange. From histological techniques to radiological approximations, all the methods that have been employed in the process have failed in reaching a consensus. In 1967, Thurlbeck reported that the area of emphysematous lungs ranged from 40 to 100 m^2^, normally averaging around 63 m^2^ [4]. Hasleton in 1972 estimated that the pulmonary surface area ranged from 23.56 to 68.76 m^2^ [5]. Weibel provided three values at three different points in time: 150-180 m^2^ in 1980, 80 m^2^ in 1993, and 130 m^2^ in 2009 [6,7]. 

The physiological estimates for the pulmonary surface area ranged from 70 m^2^ to 140 m^2^ across our literature search, but none of them matches up to the area of a tennis court. Table 1 enlists the comparisons and the provided estimate if any [2,5,8-19].

**Table 1:** Alveolar surface likened to tennis courts in medical literature.

NO	SOURCE	ANALOGY	AREA(m^2^)	REFERENCE
1	Berne & Levy physiology	The lungs are contained in a space with a volume of approximately 4 L, but they have a surface area for gas exchange that is the size of a tennis court (∼85 m2).	85	2
2		This alveolar-capillary network is composed of thin epithelial cells of the alveolus and endothelial cells of the vessels and their supportive matrix and has an alveolar surface area of about 70 m2 (about the size of a tennis court).	70	
3	Vander’s Human Physiology: The Mechanisms of Body Function	The total surface area of alveoli in contact with capillaries is roughly the size of a tennis court.		8
4	Murray & Nadel’s Textbook of Respiratory Medicine	This juxtaposition of capillaries with alveoli provides the vast surface area needed for effective gas exchange: approximately 70 m^2^ (two thirds the area of a tennis court)	70	9
5		The human lung has a large surface area, which for an average-size person approximates half a doubles tennis court, thus maximizing the approximation and apposition of capillaries to the epithelial surface.		
6		The lung epithelium has a surface area approximately the size of a tennis court and represents the largest epithelial surface in the body.		
7	Fishman’s Pulmonary Diseases and Disorders	Because this small mass of tissue is spread over an enormous area – nearly the size of a tennis court – the tissue framework of the lung must be extraordinarily delicate		5
8		To this end a very large area of contact between air and blood must be established; for the human lung it is sometimes compared with the area of a tennis court in size		
9		The preceding section considered the overall size of the gas exchanger of the entire lung to compare it with the global performance of this organ. In reality, the surface the size of a tennis court is subdivided into some 400 million gas-exchange units.		
10	Textbook of Histology	It has been estimated that the total surface area of all alveoli available for gas exchange exceeds 140 m^2 ^(the approximate floor space of an average-sized two-bedroom apartment or the size of a singles tennis court).	140	10
11	Alveolar Structure and Function	With 85-95% of the alveolar surface surrounded by capillaries, the effective diffusion membrane created (total, both lungs) approaches 80m^2^, not quite as large as the singles half of a tennis court.	80	11
12	Principles of Pulmonary Medicine	It is estimated that the adult human lung has on the order of 300 million alveoli, with a total surface area approximately the size of a tennis court.		12
13	Andreoli and Carpenter's Cecil Essentials of Medicine E-Book	The alveoli are thin-walled structures with a total surface area of about 100m^2^. This is roughly half the size of a tennis court.	100	13
14	Tennis anyone? The lungs as a new court for systemic therapy	Medication consisting of a fine particle aerosol is carried to the alveolar epithelium, whose surface area is about 100 m^2^ in adults (the size of a singles tennis court) during slow deep inhalation	100	14
15	Deconvoluting lung evolution: from phenotypes to gene regulatory networks	The pulmonary gas exchanger is characterized by a very large surface of air-blood contact, nearly the size of a tennis court in humans (120 m^2^), a very thin tissue barrier (1mm); and a large capillary blood volume (200ml in humans), all of which determines the pulmonary diffusing capacity DLO2 (Weibel 2000; Weibel and Hoppeler 2000).	120	15
16	Endothelial Cell Mechano-Metabolomic Coupling to Disease States in the Lung Microvasculature	The lung has a prominent place in the microvasculature, as its estimated capillary surface area (as defined by diameter of a vessel 10 μm or less) is roughly 50–70 m^2^, which is one-fourth the size of a tennis court	50-70	16
17	Lung Parenchymal Mechanics	The parenchymal structure is thus a huge collection of tiny and fine balloons that pack an enormous surface area (close to that of a tennis court) into the chest cavity		17
18	Lung Structure and the Intrinsic Challenges of Gas Exchange	The model for structure-function correlation of the pulmonary gas exchanger so far discussed considered the whole lung: a gas-exchanging surface the size of a tennis court in humans with a capillary network containing ~200 mL of blood.		18
19	Smaller is better—but not too small: A physical scale for the design of the mammalian pulmonary acinus	To exchange oxygen and carbon dioxide, blood and air must be brought into close contact over a large surface area, nearly the size of a tennis court, in the human lung		19

The villi

The surface area of the intestinal carpet available for absorption also has been under much debate. In 1967, Wilson first illustrated the surface area of mucosa per unit serosal length as 1.58 m^2^/m between the duodenojejunal flexure and the ileocecal valve (5.5 m). Therefore, his estimate turns out to be around 8.7 m^2^ [20]. In a discussion regarding drug absorption, Niazi stated that the intestinal surface area is about 120 m^2^ [21]. A recent review of morphometric data computed that the actual intestinal surface area is just around 32 m^2^, almost a 10-fold underestimation, and concluded that the area is half the size of a badminton court and not that of a tennis lawn [22].

Most sources in our literature search provide the range of estimates between 200 m^2^ and 400 m^2^ and have been listed in Table 2 along with the parallel drawn to tennis courts [8,23-29].

**Table 2:** Gastrointestinal surface area likened to tennis courts in medical literature.

NO	SOURCE	ANALOGY	AREA(m^2^)	REFERENCE
1	Vander’s Human Physiology: The Mechanisms Of Body Function, Thirteenth Edition	The human small intestine’s total surface area is about 250 to 300 square meters, roughly the area of a tennis court.	250-300	8
2	Textbook Of Medical Physiology	Thus, the combination of the folds of Kerckring, the villi, and the microvilli increases the total absorptive area of the mucosa perhaps 1000-fold, making a tremendous total area of 250 or more square meters for the entire small intestine—about the surface area of a tennis court.	>250	23
3	Harrison's Principles of Internal Medicine (19th Ed)	The functional surface area of the small intestine is somewhat greater than that of a doubles tennis court		24
4	Medical Physiology: A Cellular and Molecular Approach	The total surface area of the human small intestine is ~200 m2, or the surface area of a doubles tennis court	200	25
5	Cellular and Molecular Immunology E-Book	First, the combined mucosa of the small and large bowel has a total surface area of more than 200 m^2^ (the size of a tennis court), made up mostly of small intestinal villi and microvilli.	200	26
6	Avery's Diseases of the Newborn E-Book	The fully developed gastrointestinal (GI) tract reaches a total length of approximately 20 to 30 feet (Hounnou et al, 2002) and has a mucosal surface area of 300 to 400 m^2^ (DeWitt and Kudsk, 1999), which is equivalent to the size of a singles tennis court.	300-400	27
7	Intestinal crosstalk – a new paradigm for understanding the gut as the “motor” of critical illness	The mucosal surface of the gut represents the largest body surface in contact with the outside world (approximately 300 m^2^, roughly the area of a tennis court).	300	28
8	Oxygen in the regulation of intestinal epithelial transport	When one considers that the intestinal epithelium covers an area equivalent to a tennis court and that it must actively pull 9 l of fluid and approximately 1 kg of nutrients from the lumen each day, one can get an appreciation of the energy requirement for the Na^+^/K^+^-ATPase to perform its functions		29

The endothelium

The vascular endothelium is another instance of repetitive analogies to tennis courts. In 1929, Krogh estimated that the cumulative surface area summed up to 6,300 m^2^ [30]. On the other end of the spectrum, Pries et al. asserted that the blood-endothelium interface measured about 350 m^2^ [31]. Table 3 shows the endothelial surface [24,32-42].

**Table 3:** Endothelial surface likened to tennis courts in medical literature.

NO	SOURCE	ANALOGY	AREA(m^2^)	REFERENCE
1	Harrison's Principles of Internal Medicine (19th Ed)	Endothelial cells line the surface of the entire circulatory tree, totalling 1–6 × 10^13^ cells, enough to cover a surface area equivalent to about six tennis courts.		24
2	Vascular Medicine: A Companion to Braunwald's Heart Disease	The endothelium serves as the innermost lining of all blood vessels. It is the largest organ in the body weighing approximately 1.0 to 1.8 kilograms, containing approximately 1 x 10^13^ cells, and representing a surface area roughly equivalent to 6 to 8 tennis courts.		32
3	Physical Activity and Cardiovascular Disease Prevention	The vascular endothelium of an average sized individual contains approximately 10,000,000,000,000 endothelial cells that weigh 1.5 kg and covers almost 700 m^2^, an area equivalent to six tennis courts	700	33
4	Body Renewal: The Lost Art of Self-Repair	Stretched out, your endothelia might cover a tennis court		34
5	The Metabolic Syndrome	In the adult human, it represents 1 per cent of body mass with a collective surface area of 350 m^2^ (Pries, Secomb and Gaehtgens, 2000), the equivalent of approximately one and a half tennis courts.	350	35
6	The Endothelium and Endothelin: Beyond Vascular Reactivity	The vessel wall is 5 times the size of the heart in mass and 6 times the size of a tennis court in area.		36
7	Endothelial dysfunction: a comprehensive appraisal	Although it is a monolayer that covers the inner surface of the entire vascular system, its total weight is more than a liver and has a mass equal to several hearts or, if it is extended, covers a various tennis courts surface area.		37
8	Does Endothelium Buffer Fat?	The interface between ECs and plasma is between 4000 and 7000 m^2^, equivalent to the surface of >20 tennis courts.	4000-7000	38
9	Obesity and risk of vascular disease: importance of endothelium-dependent vasoconstriction	Endothelial cells form the inner lining of arterial and venous blood vessels and lymphatic vessels which amount to approximately 1.5 kg in a person weighing 70 kg, covering an area ofapproximately four tennis courts		39
10	Textbook of Vascular Medicine	The average capillary density in the body is 600 vessels/mm^3 ^tissue with around 1000m^2 ^surface area available for exchange of materials, which is equivalent to the surface area of almost four tennis courts.	1000	40
11	Endothelial Mechanotransduction, Redox Signaling and the Regulation of Vascular Inflammatory Pathways	In an adult human, the surface area of the entire endothelium is 3,000 m^2^ which is equivalent to at least six tennis courts	3000	41
12	Holland-Frei Cancer Medicine	An angiogenic focus appears as only a tiny fraction or a small “hot spot” of proliferating and migrating endothelial cells that arise from a monolayer of resting endothelium of approximately 1000 m^2^, an area the size of a tennis court.	1000	42

Discussion

**Figure 3:**
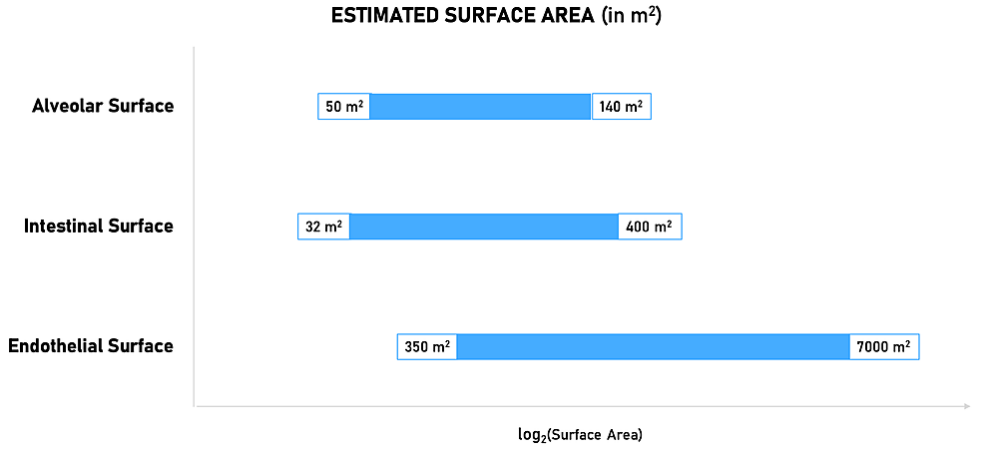
The error in the quantification of enormous surfaces in the body.

Across the literature, there is a 20-fold variation (350 m^2^ to 7,000 m^2^) in the estimated endothelial surface area and around two-fold variation in the alveolar surface area (70 m^2^ to 140 m^2^). The intestinal surface area (32 m^2^ to 400 m^2^) displays a variation 12.5 times the lowest estimate (Figure 3). Such large-scale discrepancies are unlikely to result from the variability in processing and measuring techniques or the nutritional state of the human subject. This myriad of mismeasurements is attributable to the nature of these surfaces. They are irregular patterns called fractals. 

Fractals are irregular geometric patterns. Euclidian shapes like a perfect square or a sphere are hardly ever found naturally. Whereas fractals are present everywhere in nature: from the branching of trees to river networks, the pattern of stars in the galaxy to whirlwinds. “Clouds are not spheres, mountains are not cones, and lightning does not travel in a straight line,” says Benoit Mandelbrot, the scientist who described the language of fractal geometry. Fractals are a result of a process that is iterated or repeated multiple times. They exhibit symmetry across different scales, known as self-similarity. Although the level of magnification changes, the degree of their roughness or irregularity remains fairly constant, which can be characterized by a number called the fractal dimension [43,44].

**Figure 4:**
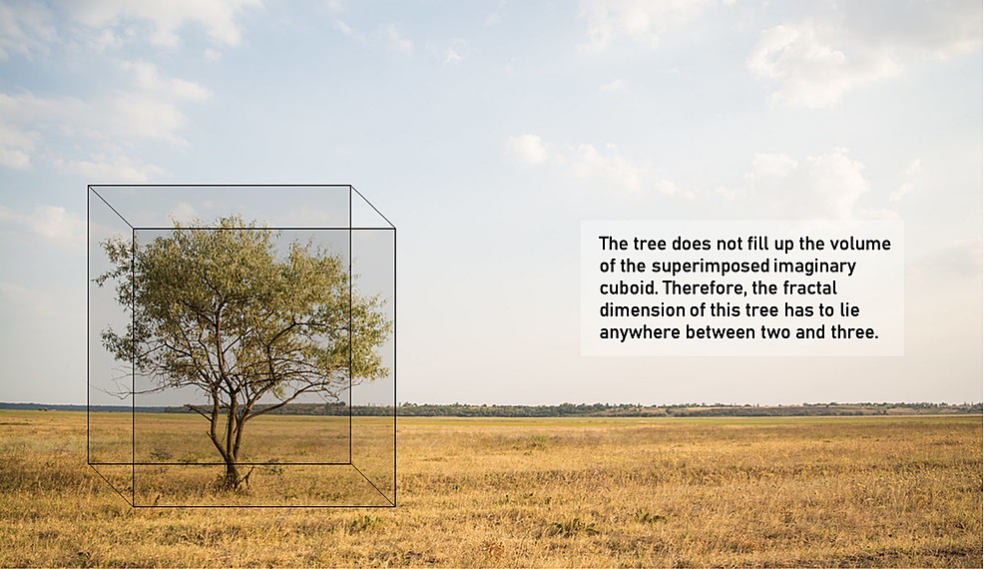
A tree is a fractal.

Understanding the concept of “dimension” is necessary before venturing into fractals or fractional dimensions. Geometry deals with “objects” and “spaces.” Therefore, the dimension of an object is distinct from the dimension of the space in which it lives or exists. The dimension of the space is the number of axes in which an object can move freely (degrees of freedom). For instance, a histological slide can be regarded as a plane with a dimension of two; the world we live in has a dimension of three. However, the dimension of an object reflects how the object fills up the space within which it exists [45]. Let us take an example of a tree (Figure 4). It exists in three-dimensional space; however, it does not completely fill up the volume in all three dimensions, like a cuboid. A tree, therefore, fills up more space than a rectangle but not as much as that of a cuboid. Thus, the dimension of the tree would lie somewhere between two and three. This can be conceptualized as the fractal dimension of the tree.

Fractals are a signature of any process that is chaotic, non-linear, and dynamic. So are all biological processes. The human body is a conglomeration of a multitude of chaotic processes occurring in synchrony, good enough to keep us alive. The branching of the tracheobronchial tree, the intricate network of blood vessels, the system of ducts collecting hormones, the retinal vasculature are some instances of geometric fractals in the body [46]. Mathematical fractals are infinite; however, biological entities display the properties of fractals within a definite “scaling window,” which is determined by physical constraints. For instance, in the tracheobronchial tree, the surface properties of the mucus limit further branching.

## Conclusions

It is challenging to explain the prevalence of the “tennis court” metaphor across medical literature, and in most cases, it is not factually based. The language of fractals is what nature understands, and it is appropriate to define natural surfaces in terms of fractal geometry. Although biological entities behave as fractals within the confines of a window, it is inappropriate to apply Euclidian geometry for quantification. Mathematical modeling, based on radiological measurements can provide a more accurate estimate of these surface areas. Nonetheless, finding the exact value in terms of accuracy is out of contention.
